# Eco-Engineered Gold Nanoparticles Via *Laurus Nobilis* at Native pH: Toward Multifunctional Nanoformulations for in Vitro Cancer Therapy

**DOI:** 10.1007/s12010-025-05561-1

**Published:** 2026-01-13

**Authors:** Bilsen Tural, Gülşah Eşlik, Erdal Ertaş, Ömer Erdoğan, Servet Tural

**Affiliations:** 1 Department of Nanotechnology, Graduate School of Natural and Applied Sciences, Dicle University, 21280 Diyarbakir, Türkiye; 2 Department of Chemistry, Graduate School of Natural and Applied Sciences, Dicle University, 21280 Diyarbakir, Türkiye; 3 Department of Chemistry Education, Ziya Gökalp Faculty of Education, Dicle University, 21280 Diyarbakir, Türkiye; 4 Department of Food Processing, Technical Sciences Vocational School, Batman University, 72060 Batman, Türkiye; 5 Department of Biochemistry, School of Medicine, Gaziantep Islam Science and Technology University, Gaziantep, 27010 Türkiye

**Keywords:** Green synthesis, Gold nanoparticles (AuNPs), *Laurus Nobilis* extract, cytotoxicity, Wound healing inhibition, plant-mediated nanotherapeutics

## Abstract

In this study, gold nanoparticles (AuNPs) were developed using *Laurus nobilis* (LN) leaf extract, and their cytotoxic and antimigratory effects were evaluated in vitro to investigate their potential in cancer treatment and wound healing. AuNPs were synthesized by green reduction of HAuCl₄ using LN extract at room temperature. Characterization was carried out by UV-Vis, TEM, SEM, EDX, FTIR and DLS. Cytotoxicity was assessed by MTT assay in four cancer cell lines: A549, MDA-MB-231, SH-SY5Y, and L929. An in vitro wound healing assay using A549 cells was conducted to assess the antimigratory effects. Statistical analysis included IC₅₀ values, effect size (Cohen’s d), AUC, and non-parametric testing. LN-AuNPs showed a sharp plasmon resonance peak (~ 540 nm), narrow size distribution (~ 69 nm), and minimal aggregation. Spectral and elemental data confirmed phytochemical-mediated reduction. LN-AuNPs significantly decreased cell viability in all tested lines, with the lowest IC₅₀ observed in L929 cells (0.02 µg/mL). All groups exhibited large effect sizes and statistical significance. Additionally, LN-AuNPs markedly inhibited cell migration in A549 cells, confirming their antimigratory potential. This is the first study to report both cytotoxic and wound-healing-inhibitory effects of LN-AuNPs. The findings highlight LN-AuNPs as promising green-synthesized nanoplatforms for integrated cancer treatment and regenerative medicine applications.

**Graphical Abstract:**

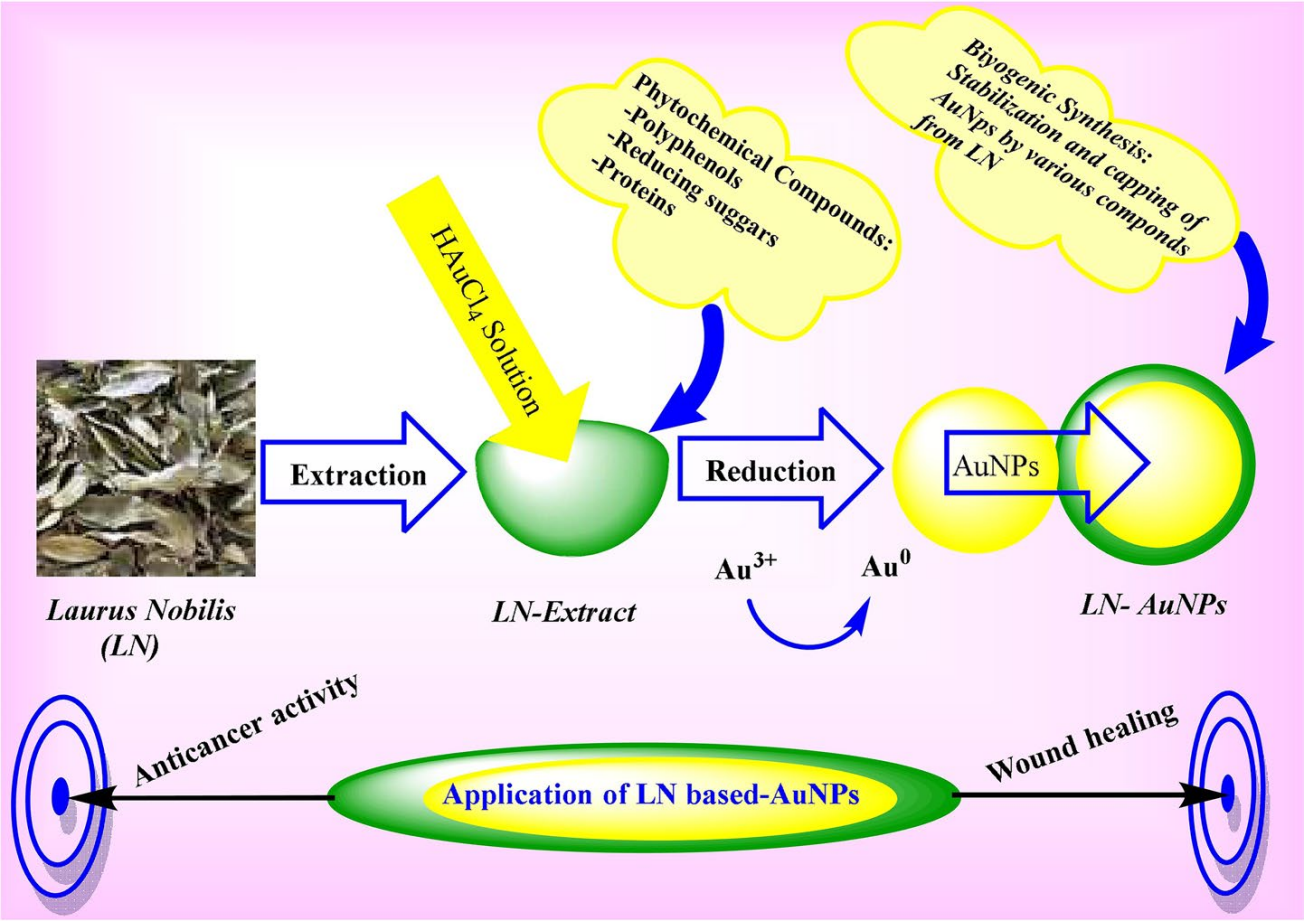

**Supplementary Information:**

The online version contains supplementary material available at 10.1007/s12010-025-05561-1.

## Introduction

Gold nanoparticles (AuNPs) are extensively studied in nanomedicine owing to their unique physicochemical properties, such as high surface-area-to-volume ratio, tunable surface plasmon resonance (SPR), biocompatibility, and ease of surface modification. These features enable their broad application in drug delivery, molecular imaging, biosensing, and cancer therapy [1, 2].

In recent years, green synthesis methods utilizing plant extracts have emerged as sustainable and bioactive alternatives to conventional physical and chemical routes. Phytochemicals such as flavonoids, terpenoids, and polyphenols function as natural reducing and stabilizing agents, resulting in biogenic nanoparticles that often exhibit synergistic therapeutic properties derived from their botanical origin [3, 4].

*Laurus nobilis* (LN), a medicinal plant native to the Mediterranean basin, is rich in essential oils, phenolic acids, and flavonoids, and has demonstrated antioxidant, anti-inflammatory, antimicrobial, and anticancer activities [5–7]. Previous studies have shown that LN leaf extract can inhibit proliferation and induce apoptosis in cancer cell lines such as MCF-7 and SH-SY5Y [8, 9]. Moreover, LN*-*derived AuNPs have been reported to possess enhanced antimicrobial activity compared to the crude extract, highlighting the therapeutic potential of phytochemical-capped nanostructures.

In addition to their anticancer effects, AuNPs have shown promise in accelerating wound healing through modulation of fibroblast migration, oxidative stress reduction, and extracellular matrix remodeling [10, 11]. These properties are often amplified when nanoparticles are functionalized with bioactive phytochemicals. Despite its historical use in topical formulations, the role of LN in nanomaterial-based regenerative medicine has not been systematically explored.

To the best of our knowledge, no previous study has simultaneously evaluated the anticancer and antimigratory effects of AuNPs biosynthesized using LN extract. In this study, we present a novel dual-functional nanoplatform LN-AuNPs and assess its cytotoxic effects across four cancer cell lines (A549, MDA-MB-231, SH-SY5Y, and L929) alongside its wound healing-inhibitory activity in A549 cells. By integrating green synthesis, multi-instrumental characterization, and in vitro bioactivity assays, this work advances the biomedical potential of plant-mediated nanotechnology toward multifunctional therapeutic systems.

## Materials and Methods

### Materials

Chloroauric acid trihydrate (HAuCl₄·3 H₂O, ≥ 99.9%) was purchased from Sigma-Aldrich (USA). Dulbecco’s Modified Eagle Medium (DMEM), fetal bovine serum (FBS), phosphate-buffered saline (PBS), trypsin–EDTA, and MTT reagent were obtained from Gibco (Thermo Fisher Scientific, USA). All chemicals were of analytical grade and used without further purification.

### ***Preparation of*** LN ***Leaf Extract***

Fresh LN leaves were collected from a local source, thoroughly washed with distilled water, and shade-dried for seven days. The dried material was ground into a fine powder using a mechanical grinder. Ten grams of powdered leaves were boiled in 100 mL of distilled water at 60 °C for 30 min. The mixture was filtered through Whatman No. 1 filter paper, and the filtrate was stored at 4 °C for subsequent use [12].

### Green Synthesis of LN-AuNPs

To synthesize LN-AuNPs, 10 mL of LN extract was added dropwise to 90 mL of 1 mM aqueous HAuCl₄ solution under constant magnetic stirring at room temperature. The development of a ruby red color indicated the formation of AuNPs. The reaction was allowed to proceed for 24 h, followed by centrifugation at 10,000 rpm for 15 min. The resulting pellet was washed with distilled water and ethanol and redispersed in deionized water [12].

To investigate pH effects, the optimized formulation (1 mM HAuCl₄ + 30% extract) was resynthesized at pH 3, 7, and 11, in addition to the natural pH of the extract (unaltered). The nanoparticles synthesized at natural pH were used for all biological experiments based on their superior physicochemical stability.

### Characterization

The optical properties of the LN-AuNPs were characterized using UV–Vis spectroscopy (Agilent Cary 60, USA). Functional groups involved in reduction and capping were identified by FTIR spectroscopy (Agilent Cary 630, USA). Transmission Electron Microscopy (TEM; JEOL JEM-2100, Japan) and Scanning Electron Microscopy (SEM; QUANTA 400 F, USA) were utilized to examine the size and surface morphology of nanoparticles, with elemental mapping performed via Energy Dispersive X-ray Spectroscopy (EDX). Dynamic light scattering (DLS) and zeta potential measurements were performed using a Malvern Zetasizer Nano ZS (Malvern Instruments, UK**)** to evaluate hydrodynamic diameter and colloidal stability.

### Cell Culture

Human lung carcinoma (A549), breast adenocarcinoma (MDA-MB-231), neuroblastoma (SH-SY5Y), and mouse fibrosarcoma (L929) cell lines were obtained from the American Type Culture Collection (ATCC). Cells were cultured in DMEM supplemented with 10% FBS, 1% penicillin–streptomycin, 2 mM L-glutamine, and 10 mM HEPES. Cultures were maintained at 37 °C in a humidified incubator with 5% CO₂ and subcultured at 80–90% confluence. Experiments were performed using cells between passages 5 and 20 [13].

### MTT Cytotoxicity Assay

Cytotoxicity was assessed using the MTT assay. Cells were seeded in 96-well plates at a density of 5 × 10³ cells/well and incubated for 24 h. Cells were then treated with increasing concentrations (1, 10, 100, and 1000 µg/mL) of either LN extract or LN-AuNPs for 24 h. Following treatment, 10 µL of MTT solution (5 mg/mL) was added to each well and incubated for 4 h. The medium was discarded, and the resulting formazan crystals were dissolved in 100 µL of dimethyl sulfoxide (DMSO). Absorbance was measured at 570 nm using a BioTek ELx800 microplate reader [14]. Cell viability was calculated using the formula (1):

1 $$Cell\;viability\;(\%)=\left(Abs_{sample}/Abs_{control}\right)\times100$$

### Wound Healing (Scratch) Assay

To assess the effect on cell migration, a scratch assay was performed using A549 cells. Cells were seeded in 12-well plates (1 × 10⁵ cells/well) and cultured to confluence. A scratch was made in the monolayer using a sterile 200 µL pipette tip. After washing with PBS, cells were treated with LN or LN-AuNPs at their respective IC₅₀ concentrations. Images were taken at 0 and 24 h using an inverted microscope (Olympus CKX41). Wound closure was quantified using ImageJ software (NIH), and results were expressed as a percentage reduction in wound width [14].

### Statistical Analysis

All experiments were performed in biological triplicates (*n* = 3), and results are presented as mean ± standard deviation (SD). Statistical analysis was performed using GraphPad Prism 9.3.0 (GraphPad Software, USA). Normality was assessed using the Shapiro–Wilk test, and variance homogeneity was evaluated using Levene’s test. Comparisons between LN and LN-AuNPs at each concentration were made using two-tailed Student’s t-tests. IC₅₀ values and Hill slopes were derived from four-parameter logistic nonlinear regression models. Area under the curve (AUC) was calculated using the trapezoidal rule.

Effect sizes were computed using Cohen’s d and interpreted as small (0.2–0.5), medium (0.5–0.8), or large (> 0.8). Kruskal–Wallis tests followed by Mann–Whitney U tests were applied to evaluate differences in sensitivity among cell lines. Wound healing data were also analyzed using the Mann–Whitney U test, and effect sizes were reported. Statistical significance was set at *p* < 0.05. In all figures, significance levels are indicated as follows: *p* < 0.05 (*)*,*p* < 0.01 (***)***, *p* ***<*** 0.001, and *p* < 0.0001 (****).

## Results

### Optimal SPR Response Achieved with Moderate Extract and Low Precursor Concentration

**Fig. 1:**
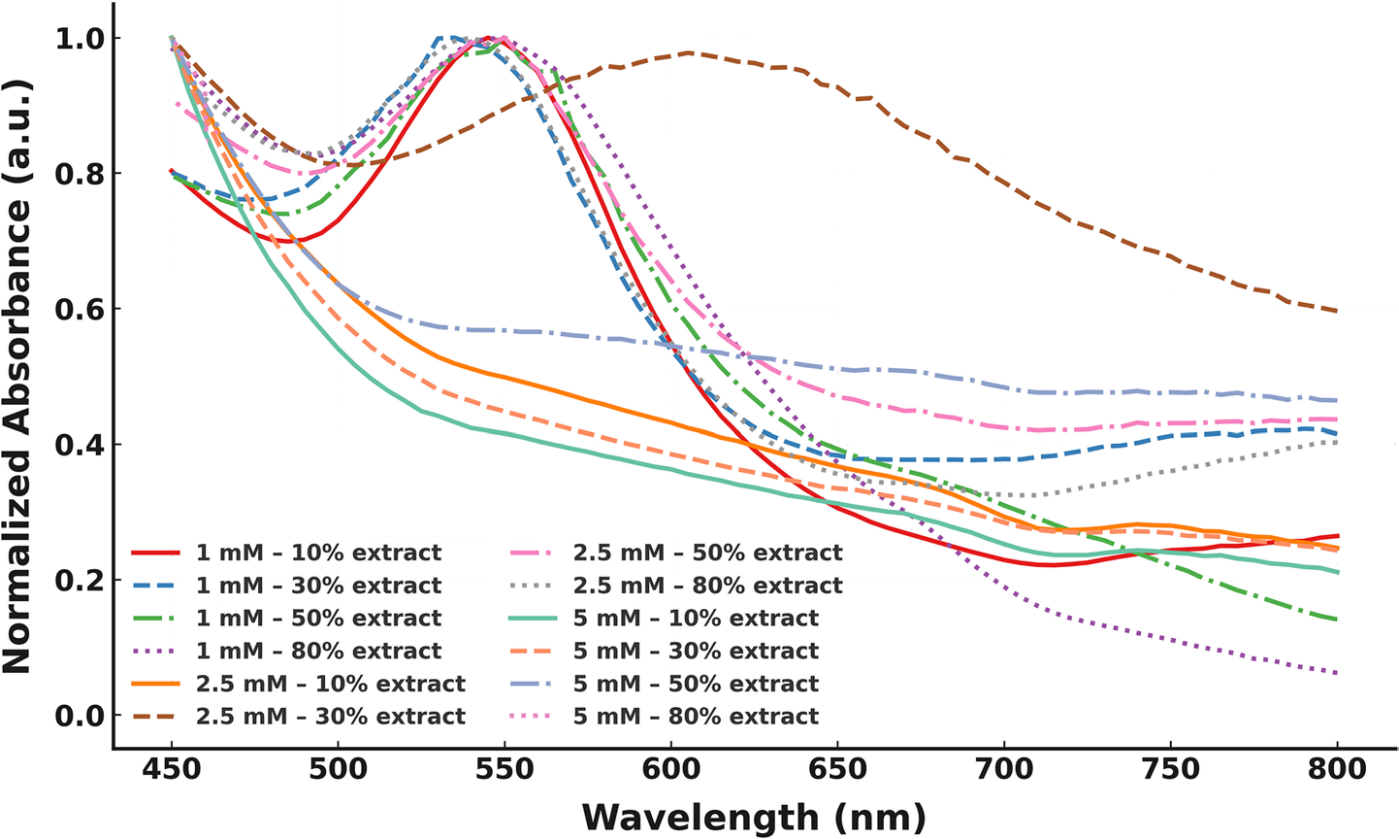
UV–Vis spectra of AuNPs synthesized using varying concentrations of LN extract (10–80%) and HAuCl₄ (1, 2.5, and 5 mM)

AuNPs synthesis using LN extract was confirmed by UV–Vis spectroscopy. All formulations exhibited characteristic surface plasmon resonance (SPR) bands between 520 and 580 nm (Fig. 1), verifying successful nanoparticle formation. The sharpest and most intense SPR peaks were observed in the 1 mM HAuCl₄ group, particularly at 30–50% extract ratios, indicating optimal nucleation and reduction conditions [15]. In contrast, 5 mM precursor concentrations yielded broadened or dampened peaks, suggestive of polydispersity or aggregation.

**Fig. 2:**
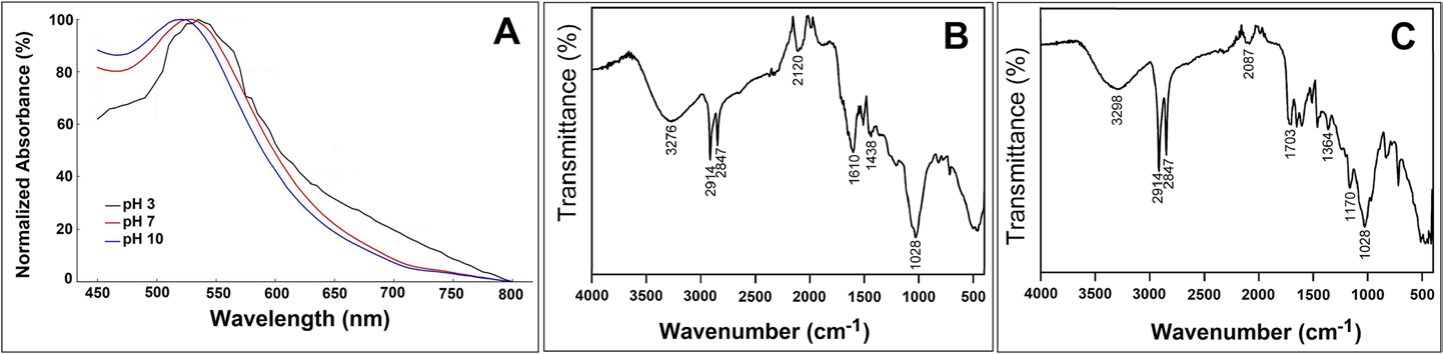
(**A**) Normalized UV–Vis spectra of AuNPs synthesized at pH 3, 7, and 10, showing red-shifted and sharper SPR peak at pH 3. (**B**) FTIR spectrum of crude LN extract indicating characteristic O–H, C–H, and C = O peaks. (**C**) FTIR spectrum of LN-AuNPs after synthesis showing shifts indicative of phytochemical binding and reduction

When normalized spectra were compared across pH values (Fig. 2A), the synthesis at pH 3 showed a red-shifted and sharper SPR peak (~ 540–550 nm), consistent with formation of smaller and more monodisperse particles. In contrast, alkaline conditions (pH 10) led to broader and less defined peaks. These findings align with previous reports showing that acidic pH favors nucleation and monodispersity in green synthesis reactions [16].

### FTIR Confirms Polyphenol Involvement in Reduction and Stabilization

FTIR analysis (Fig. 2B, C) of the extract and LN-AuNPs revealed shifts in characteristic peaks, indicating the involvement of phytochemicals in reduction and stabilization. The extract exhibited prominent O–H (3298 cm⁻¹), C–H (2914 and 2847 cm⁻¹), C = O (1703 cm⁻¹), and C–O (1028 cm⁻¹) stretches. After nanoparticle formation, notable shifts were observed, including a shift in the O–H peak to 3276 cm⁻¹ and a new band at 2120 cm⁻¹, suggesting complexation and reduction by phenolic and carboxyl groups [17].

### TEM Reveals Predominantly Spherical Nanoparticles with Narrow Size Range

**Fig. 3:**
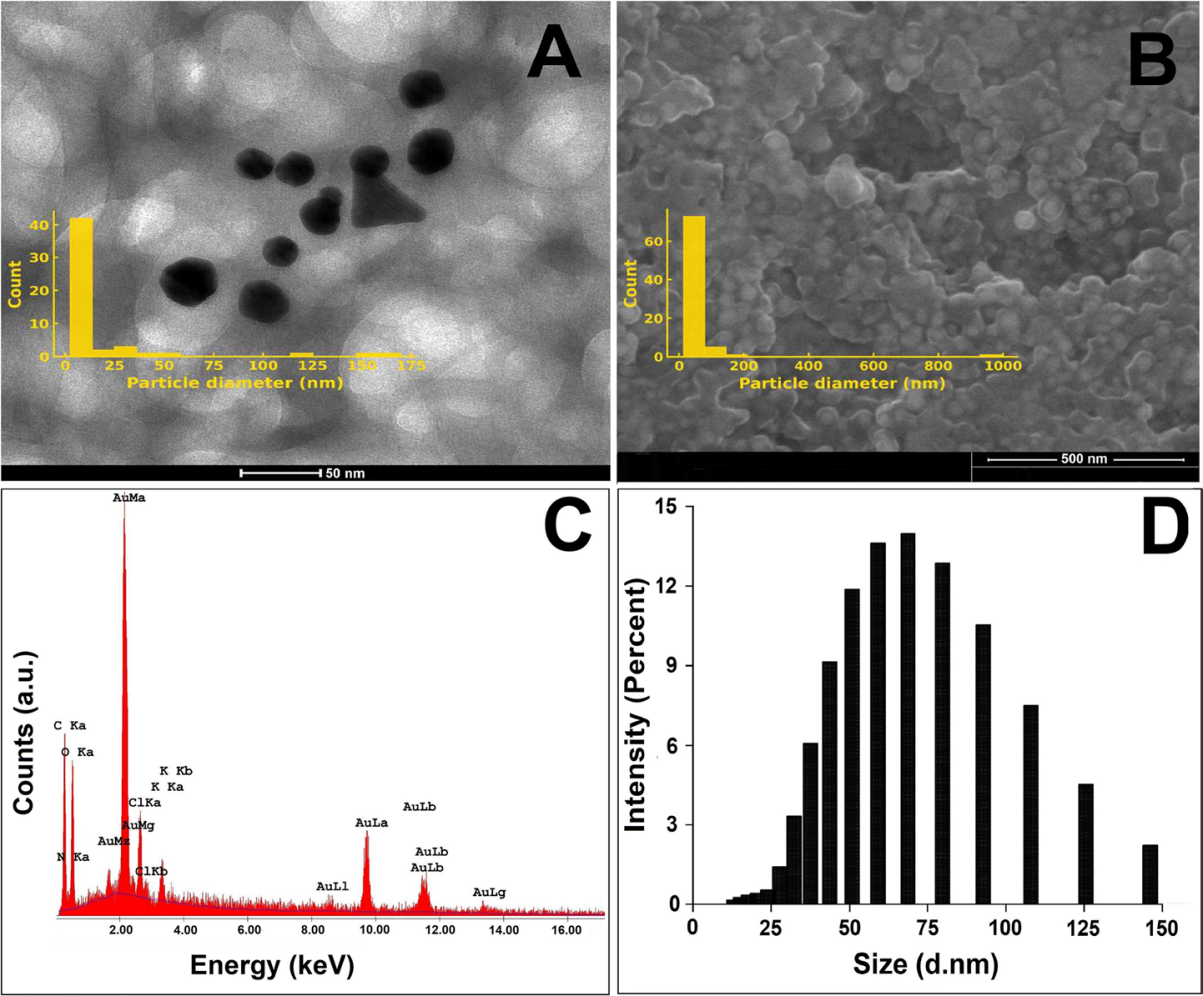
Morphological and compositional properties of LN-AuNPs (**A**) TEM image showing predominantly spherical LN-AuNPs synthesized with 30% LN extract and 1 mM HAuCl₄; occasional triangular and polygonal shapes were observed. The inset histogram represents the particle size distribution obtained from ImageJ analysis of TEM images. (**B**) SEM image showing densely packed, roughly spherical LN-AuNPs with moderate size uniformity. (**C**) EDX spectrum confirming elemental gold (Au) along with carbon (**C**) and oxygen (O) attributed to phytochemical capping. (**D**) Dynamic light scattering (DLS) profile showing hydrodynamic size distribution of LN-AuNPs synthesized at native pH

TEM images (Fig. 3A) confirmed that the majority of LN-AuNPs were spherical, with sizes ranging from 20 to 50 nm, as illustrated by the inset size distribution histogram. Occasionally, triangular and polygonal shapes were also observed, reflecting the diverse phytochemical composition of LN and its influence on crystal growth [18, 19]. The histogram analysis further indicated a narrow size distribution, supporting the efficient capping role of the plant extract and the uniform particle morphology.

### SEM Shows Uniformly Distributed Particles with Dense Surface Coverage

SEM micrographs (Fig. 3B) showed dense, uniformly distributed spherical LN-AuNPs with minimal aggregation. The inset histogram derived from SEM measurements demonstrated a relatively consistent particle size distribution, while the observed surface texture and compact arrangement suggest efficient stabilization and homogeneous particle formation, consistent with prior reports using *Terminalia catappa* and *Hibiscus rosa-sinensis* extracts [20, 21].

### EDX Confirms Elemental Au and Organic Shell Composition

EDX analysis (Fig. 3C) revealed a strong Au peak (~ 2.1 keV), alongside carbon (38.04%) and oxygen (17.20%), indicating phytochemical capping. Trace levels of nitrogen, chlorine, and potassium were also detected. The relatively high Au purity (~ 39%) and absence of foreign metals confirm the green and clean synthesis profile [22].

### Smallest and Most Stable Particles Formed at Natural pH

Dynamic light scattering (DLS) analysis showed that the smallest average particle size occurred under native pH conditions (68.69 ± 0.99 nm), followed by synthesis at pH 7 (70.44 ± 0.69 nm). Particles synthesized at pH 3 and 11 were significantly larger (see Fig. 3D and Supplementary Table S1). Zeta potential measurements, presented in Supplementary Table S1, indicated that nanoparticles synthesized at native pH and pH 7 exhibited the most negative surface charge (− 18.14 to − 18.80 mV), suggesting strong electrostatic stabilization.

### LN-AuNPs Exhibit Stronger Cytotoxicity than LN across all Cell Lines

**Fig. 4:**
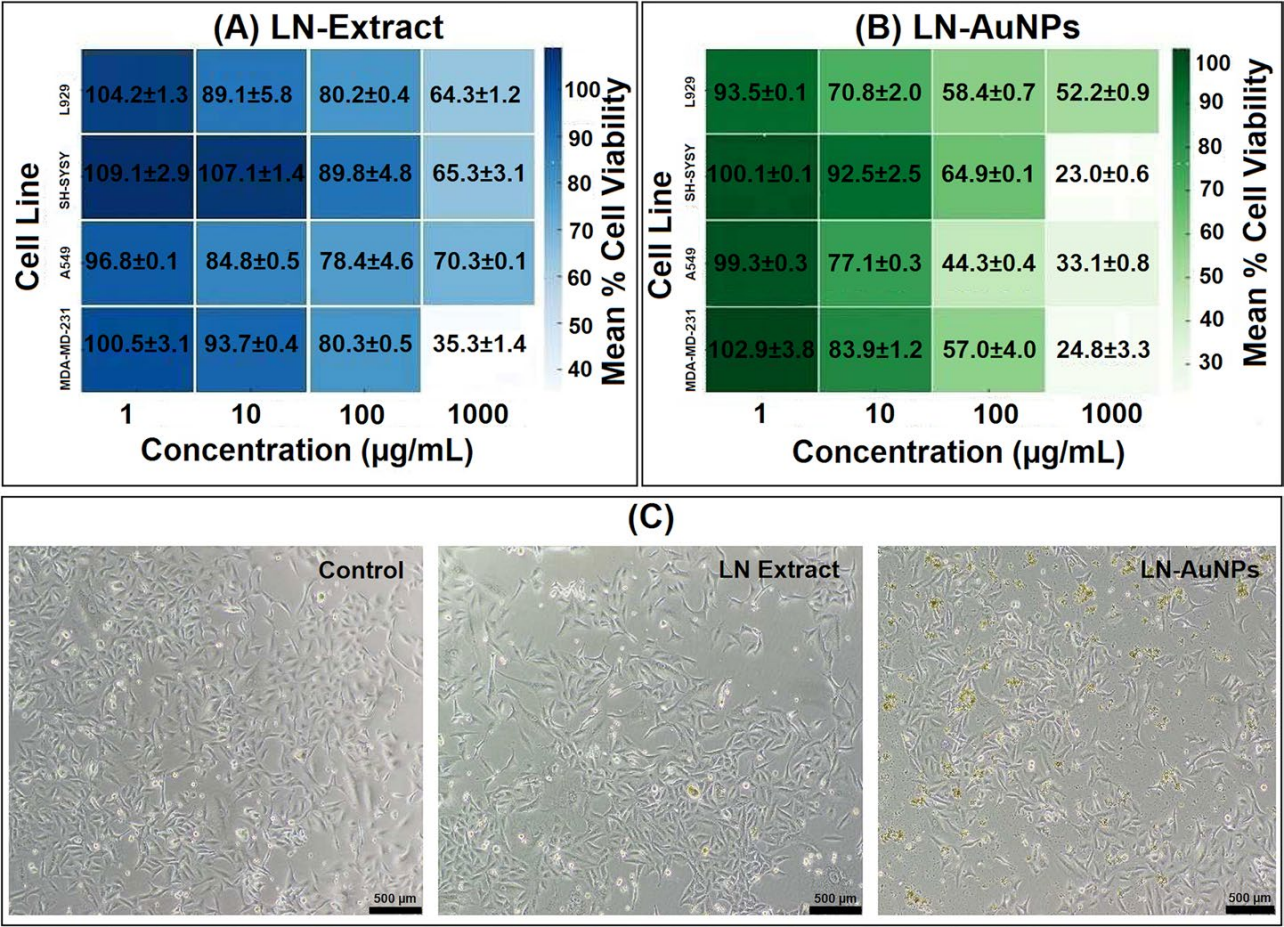
Cytotoxic and morphological effects of LN and LN-AuNPs. (**A**) Heatmap of % cell viability in four cancer cell lines after treatment with LN extract at 1, 10, 100, and 1000 µg/mL. (**B**) Heatmap of cell viability after treatment with LN-AuNPs at equivalent doses. Values are presented as mean ± SD; no statistically significant differences were found between groups at any dose level. (**C**) Representative images of A549 cells after 24 h treatment. Control cells show typical morphology; LN-treated cells exhibit minimal change; LN-AuNPs-treated cells display rounding and detachment

MTT assays showed that LN-AuNPs induced significantly greater cytotoxicity than the crude LN extract across all cell lines (L929, SH-SY5Y, A549, and MDA-MB-231) (see also Supplementary Table S2 and Fig. 4A, B). The most pronounced effects were observed in L929 and SH-SY5Y cells. Microscopy (Fig. 4C) revealed signs of apoptosis such as shrinkage and detachment in LN-AuNP-treated groups, further confirming cytotoxic action.

### IC₅₀ and AUC Values Reveal Potent Anticancer Activity, Especially in L929 Cells

Four-parameter logistic modeling revealed that L929 cells were most sensitive (IC₅₀ = 0.02 µg/mL), followed by A549 (17.95 µg/mL), while SH-SY5Y and MDA-MB-231 were more resistant (Table 1). Hill slopes and AUC values supported these findings, with steeper curves and greater overall potency in L929.

**Table 1:** Integrated analysis of Dose–Response parameters and cytotoxic potency (AUC) of LN-AuNPs across human cancer cell lines

Cell Line	IC_50_ (µg/mL)	Hill Slope	Bottom Asymptote	Top Asymptote	*R*² (Goodness of Fit)	AUC (LN-AuNPs)
L929	0.020	0.340	273.38	46.53	1.000	56311.63
SH-SY5Y	226.050	0.740	101.94	−3.12	1.000	47492.04
A549	17.950	0.880	104.63	31.05	1.000	41099.82
MDA-MB-231	933.490	0.280	130.50	−78.86	1.000	44024.28

#### Large Effect Sizes Confirm Robust Separation between Treatment Groups

Cohen’s d effect size analysis (Table 2) showed consistently large values (d > 0.8) across most comparisons. Particularly high values were observed at 100–1000 µg/mL in L929 and A549, indicating substantial treatment effects and minimal overlap with control groups.

**Table 2:** Integrated summary of Dose–Response modeling and effect size analysis for LN-AuNPs across human cancer cell lines

Cell Line	Dose (µg/mL)	Cohen’s d	Effect Size Interpretation (per Cohen’s guidelines)
L929	1	11.69	Large
	10	4.13	Large
	100	34.12	Large
	100	10.66	Large
SH-SY5Y	1	4.41	Large
	10	6.11	Large
	100	7.29	Large
	100	18.63	Large
A549	1	−9.39	Large
	10	18.10	Large
	100	10.39	Large
	100	51.31	Large
MDA-MB-231	1	−0.59	Medium
	10	8.93	Large
	100	6.64	Large
	100	3.34	Large

#### Statistical Analysis Shows Differential Sensitivity among Cancer Cell Lines

**Fig. 5:**
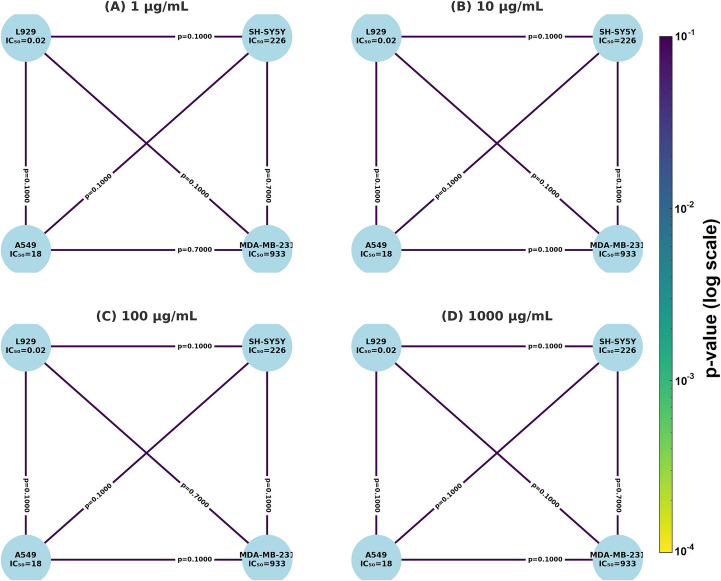
Network diagrams showing pairwise Mann–Whitney U test comparisons (**A**–**D**) Cytotoxic comparisons among L929, SH-SY5Y, A549, and MDA-MB-231 cell lines treated with LN-AuNPs at 1, 10, 100, and 1000 µg/mL, respectively. IC₅₀ values are shown in nodes; p-values are displayed on connecting lines. Kruskal–Wallis analysis showed significance at higher doses (see Supplementary Table S3), but no individual comparison reached significance (*p* > 0.05)

Kruskal–Wallis tests revealed significant differences among cell lines at 10, 100, and 1000 µg/mL (*p* < 0.05, Table 3). Although subsequent pairwise comparisons (Mann–Whitney U) did not reach significance, network plots (Fig. 5(A-D)) illustrate the comparative sensitivity landscape across cell types.

**Table 3:** Kruskal–Wallis test results comparing cytotoxic responses of LN-AuNPs across cancer cell lines at different concentrations

Dose (µg/mL)	Kruskal-Wallis H	*p*-value
1	7.615	0.0547
10	10.385	0.0156
100	9.462	0.0237
1000	9.462	0.0237

#### LN-AuNPs Significantly Inhibit A549 Cell Migration in Scratch Assay

**Fig. 6:**
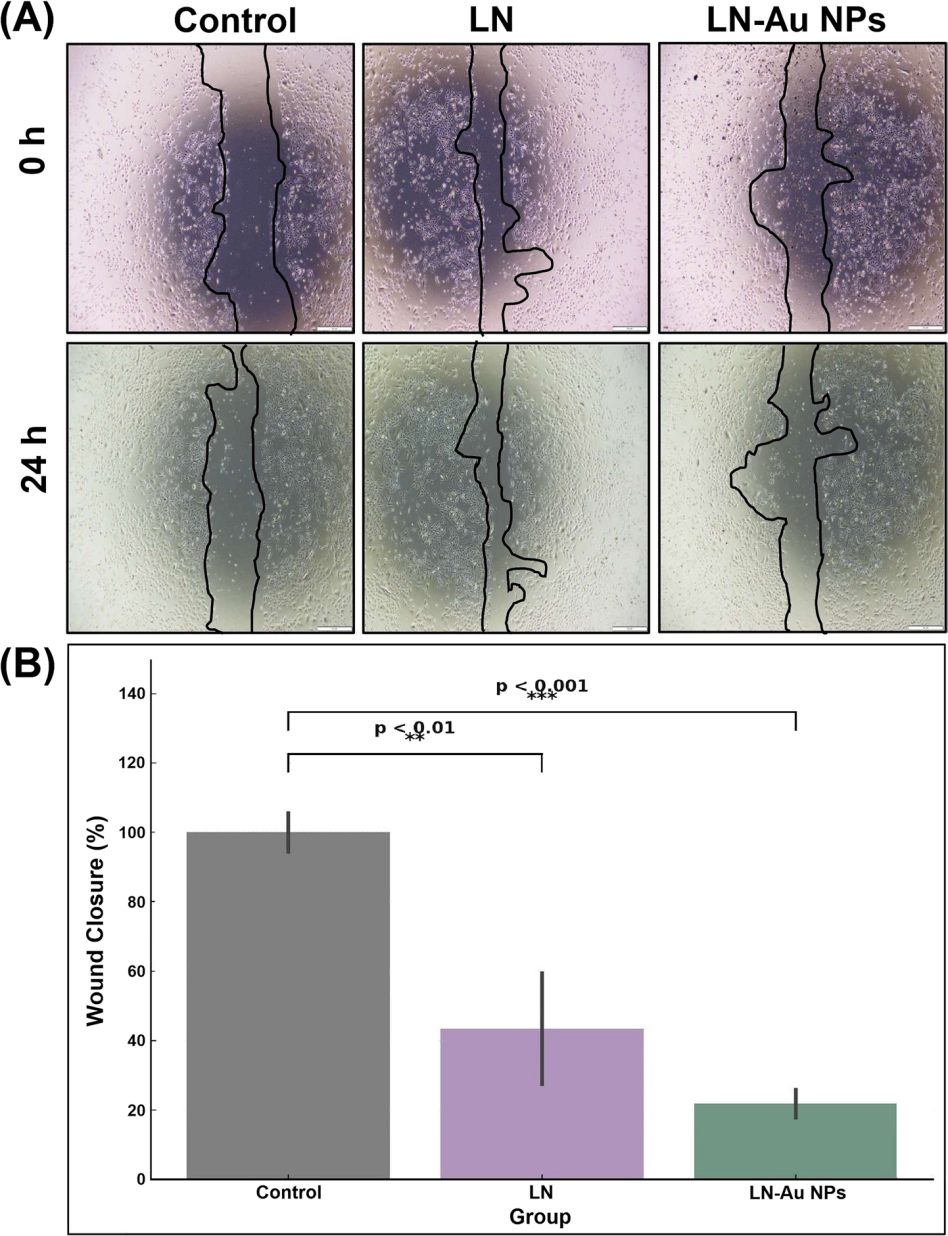
Wound healing inhibition in A549 cells (**A**) Representative scratch assay images at 0 h (top row) and 24 h (bottom row) for Control, LN-treated, and LN-AuNPs-treated groups. Wound closure was notably suppressed in the LN-AuNPs group. (**B**) Quantitative wound closure analysis. One-way ANOVA with Tukey post hoc test was used for statistical significance (*p < 0.05, **p < 0.01, ***p < 0.001 vs. control)

Scratch assay results (Fig. 6) showed impaired wound closure in both treatment groups after 24 h, with LN-AuNPs producing significantly lower closure rates than LN or control. In Fig. 6A, each group is presented with paired images captured at 0 h (top row) and 24 h (bottom row), clearly illustrating the suppressed migratory capacity over time. Effect size analysis confirmed strong antimigratory activity (Cohen’s d = 13.18 for LN-AuNPs vs. control; d = 3.86 for LN vs. control, Table 2).

## Discussion

### SPR Tuning Via Precursor and pH Control Enables Monodisperse LN-AuNP Formation

The optical behavior of LN-AuNPs was strongly influenced by synthesis parameters. UV–Vis spectra (Figs. 1 and 2A) showed that 1 mM HAuCl₄ and 30–50% extract ratios resulted in sharp SPR bands, indicating well-dispersed and monodisperse particles. This finding is consistent with previous studies where controlled metal ion availability enhanced nucleation and limited uncontrolled growth [23]. Acidic pH (pH 3) led to red-shifted and sharper SPR peaks, suggesting the formation of smaller and more uniform particles. Proton-mediated electron transfer from phenolic groups in the extract may underlie this phenomenon [24, 25].

### Biogenic Capping Guides Uniform Nanoparticle Morphology and Dispersion

TEM and SEM analyses (Fig. 3A–B) revealed predominantly spherical LN-AuNPs with occasional triangular or polygonal shapes. Such morphological diversity is typical in green syntheses and reflects the complexity of phytochemical–metal ion interactions [26–28]. The uniform surface distribution and lack of aggregation observed via SEM suggest effective capping by polyphenols and flavonoids, similar to findings in plant-based AuNPs [29, 30].

### EDX and FTIR Confirm Phytochemical Coating and Clean Synthesis

EDX analysis (Fig. 3C) confirmed the predominance of elemental Au (~ 39%) and the presence of organic capping agents (C, O, N). No toxic elements were detected, supporting the biocompatibility of the formulation. FTIR spectra (Fig. 2B, C) corroborated these results by revealing peak shifts (e.g., O–H, C = O) and new bands indicative of phenolic binding and reduction events [31, 32]. These findings are consistent with previous reports on *Camellia sinensis*- and *Cinnamomum camphora*-mediated nanoparticle syntheses. These findings are consistent with previous reports on *Camellia sinensis*- and *Cinnamomum camphora*-mediated nanoparticle syntheses [33, 34].

### Native pH Promotes Stability and Uniform Growth

Hydrodynamic size analysis based on DLS results (Fig. 3D) indicated that nanoparticles synthesized at the natural extract pH showed the smallest size (68.69 ± 0.99 nm). Zeta potential values confirming colloidal stability are presented in Supplementary Table S1. This result underscores the importance of avoiding pH modification, which can compromise the ionization state of capping ligands and result in aggregation or instability [35, 36].

### LN-AuNPs Exhibit Enhanced Cytotoxicity Compared To Crude Extract

MTT assay results (Fig. 4A, B) demonstrated significantly enhanced cytotoxicity of LN-AuNPs versus crude extract across all cell lines. The most profound effect was observed in L929 cells (IC₅₀ = 0.02 µg/mL), likely due to increased cellular uptake and prolonged retention of phytochemical-loaded AuNPs [37, 38]. The dual contribution of LN and nanoscale delivery appears to amplify therapeutic efficacy. Additional comparative statistics are summarized in Supplementary Table S2.

#### Cancer-Type-Specific Variability Reflects Resistance Mechanisms

Cell lines such as MDA-MB-231 and SH-SY5Y showed relatively higher resistance, with elevated IC₅₀ values (Table 2; Fig. 4A, B). These findings may be attributed to inherent drug resistance mechanisms, including high antioxidant capacity or efflux transporter expression, particularly in triple-negative breast cancer and neuroblastoma models [39]. Pairwise comparisons between cell lines further confirmed this trend, although most did not reach statistical significance (Supplementary Table S3 and Fig. 5).

### Effect Sizes Validate Treatment Potency Despite Sample Constraints

Cohen’s d analysis indicated exceptionally large effect sizes (up to > 50) in multiple comparisons (Table 2). Although the small sample size (*n* = 3) may contribute to statistical inflation, the biological separation between groups remains clear and robust. Similar trends have been noted in other studies with high-uptake nanoparticle systems [40].

### LN-AuNPs Inhibit Cancer Cell Migration: Antimetastatic Potential

The scratch assay revealed that LN-AuNPs significantly inhibited A549 cell migration. The antimigratory effect (Cohen’s d = 13.18 for LN-AuNPs vs. control; d = 3.86 for LN vs. control) is visually supported by Fig. 6, which includes both 0 h and 24 h images under each condition. This supports the hypothesis that LN-AuNPs not only limit proliferation but also impair key processes involved in metastasis, such as MMP activity and integrin signaling [40, 41]. The observed antimigratory activity of LN-AuNPs may be attributed to phytochemical-induced modulation of cytoskeletal architecture or inhibition of migration-associated signaling pathways. Previous studies have demonstrated that AuNPs can interfere with focal adhesion kinase (FAK) and matrix metalloproteinases (MMP-2 and MMP-9), both of which play central roles in cellular migration and invasion [42, 43]. Moreover, reactive oxygen species (ROS) generation has been proposed as a mechanism by which AuNPs induce cytoskeletal disruption and suppress epithelial-to-mesenchymal transition (EMT) in cancer cells [40]. While these mechanistic pathways were not directly investigated in this study, future work may explore the molecular basis of LN-AuNPs antimigratory effects through targeted assays including immunofluorescence or Western blotting of EMT and migration-related markers.

### Biomedical Implications and Future Outlook

Together, these findings establish LN-AuNPs as a dual-functional nanotherapeutic candidate, combining potent cytotoxic (Fig. 4; Table 2) and antimigratory activity (Fig. 6). The green synthesis route adds scalability and ecological safety. Future work should include mechanistic in vitro studies, in vivo validation, and functionalization strategies to target specific cancer types.

## Conclusions

In this study, we successfully demonstrated the biogenic synthesis of LN-AuNPs using LN extract as a dual-function nanoplatform with both anticancer and antimigratory potential. The phytochemical-rich extract not only served as a natural reducing and stabilizing agent but also imparted enhanced bioactivity to the resulting nanoparticles.

Comprehensive physicochemical analyses confirmed the formation of predominantly spherical, monodisperse LN-AuNPs with high colloidal stability and clean elemental profiles. The optimal formulation was achieved under native pH conditions using 1 mM HAuCl₄ and 30–50% extract, producing well-defined SPR peaks and minimal aggregation.

Biologically, LN-AuNPs exhibited significantly greater cytotoxic effects than crude LN extract across four cancer cell lines, particularly in L929 and A549 models, with IC₅₀ values as low as 0.02 µg/mL. Furthermore, LN-AuNPs markedly inhibited A549 cell migration in wound healing assays, indicating a strong antimigratory effect. Statistical analyses, including dose–response modeling, Cohen’s d, and AUC, reinforced the robustness and magnitude of these effects.

To our knowledge, this is the first report to integrate both anticancer and wound healing-inhibitory effects of LN-AuNPs. These findings not only highlight the therapeutic potential of LN-AuNPs as a plant-based, dual-functional nanotherapeutic system but also expand the biomedical applications of green nanotechnology. Future research should focus on in vivo validation, mechanistic pathway analysis, and formulation optimization for clinical translation.

In addition, the study underscores the broader implications of eco-engineered nanomaterials as sustainable and biocompatible alternatives to conventional chemical synthesis routes, supporting the transition toward greener biomedical technologies. Future research should focus not only on in vivo validation and mechanistic pathway analysis but also on large-scale synthesis optimization, pharmacokinetic assessments, and targeted delivery strategies to facilitate clinical translation.

## Supplementary Information

Below is the link to the electronic supplementary material.

Supplementary Material 1 (DOCX 27.3 KB)

## Data Availability

All data are given in the manuscript.
