# Supplementary material for: Eco-Engineered Gold Nanoparticles Via Laurus Nobilis at Native pH: Toward Multifunctional Nanoformulations for in Vitro Cancer Therapy
Source: Appl Biochem Biotechnol. 2026 Jan 13;198(4):2307–24. doi: 10.1007/s12010-025-05561-1 (PMC13032968; doi:10.1007/s12010-025-05561-1)
Supplement: Supplementary file 1 — Supplementary Material 1 (DOCX 27.3 KB) [file 12010_2025_5561_MOESM1_ESM.docx]

**Supplementary Materials**

**Title of the Article:** **Eco-Engineered Gold Nanoparticles via**Laurus nobilis**at Native pH: Toward Multifunctional Nanoformulations for** in vitro Cancer Therapy

**Journal:**  *Applied Biochemistry and Biotechnology*

**Authors:**
Bilsen Tural¹²³*, Gülşah Eşlik¹, Erdal Ertaş⁴, Ömer Erdoğan⁵, Servet Tural¹²³

**Affiliations:**
¹Department of Nanotechnology, Graduate School of Natural and Applied Sciences, Dicle University, 21280 Diyarbakir, Türkiye
²Department of Chemistry, Graduate School of Natural and Applied Sciences, Dicle University, 21280 Diyarbakir, Türkiye
³Department of Chemistry Education, Ziya Gökalp Faculty of Education, Dicle University, 21280 Diyarbakir, Türkiye
⁴Department of Food Processing, Technical Sciences Vocational School, Batman University, 72060 Batman, Türkiye
⁵Department of Biochemistry, School of Medicine, Gaziantep Islam Science and Technology University, 27010, Gaziantep, Türkiye

**Corresponding author:** Bilsen Tural – btural@dicle.edu.tr

**Supporting Table S1.**

**Hydrodynamic diameter and zeta potential of LN-AuNPs under different pH conditions.**
Mean ± standard deviation (n = 3). The “Natural” condition corresponds to the unadjusted pH of the *Laurus nobilis* extract-based reaction mixture.

| pH | Size (nm) | Zeta Potential (mV) |
| --- | --- | --- |
| 3 | 362.93 ± 10.67 | −10.76 ± 0.47 |
| 7 | 70.44 ± 0.69 | −18.80 ± 0.90 |
| 11 | 195.13 ± 3.46 | −16.95 ± 0.92 |
| Natural | 68.69 ± 0.99 | −18.14 ± 1.09 |

**Supporting Table S2.**

**Comparative cytotoxicity analysis of *Laurus nobilis* (LN) extract and LN-mediated gold nanoparticles (LN-AuNPs) in four human cancer cell lines.**

Percentage cell viability values (mean ± SD, n = 3) were compared using unpaired two-tailed t-tests. Statistically significant p-values (p < 0.05) are bolded. LN-AuNPs exhibited enhanced cytotoxicity across most cell lines and concentrations, with minor exceptions noted at 1 µg/mL for A549 and MDA-MB-231.

| Cell Line | Dose (µg/mL) | Test Used | p-value | LN Mean | LN-AuNPs Mean | Direction |
| --- | --- | --- | --- | --- | --- | --- |
| L929 | 1 | t-test | 0.0001 | 104.17 | 93.46 | LN-AuNPs more effective |
|  | 10 | t-test | 0.0072 | 89.14 | 70.85 | LN-AuNPs more effective |
|  | 100 | t-test | <0.0001 | 80.16 | 58.41 | LN-AuNPs more effective |
|  | 1000 | t-test | 0.0002 | 64.27 | 52.16 | LN-AuNPs more effective |
| SH-SY5Y | 1 | t-test | 0.0057 | 109.11 | 100.11 | LN-AuNPs more effective |
|  | 10 | t-test | 0.0017 | 107.08 | 92.54 | LN-AuNPs more effective |
|  | 100 | t-test | 0.0009 | 89.79 | 64.87 | LN-AuNPs more effective |
|  | 1000 | t-test | <0.0001 | 65.26 | 23.00 | LN-AuNPs more effective |
| A549 | 1 | t-test | 0.0003 | 96.77 | 99.28 | LN more effective |
|  | 10 | t-test | <0.0001 | 84.79 | 77.13 | LN-AuNPs more effective |
|  | 100 | t-test | 0.0002 | 78.38 | 44.31 | LN-AuNPs more effective |
|  | 1000 | t-test | <0.0001 | 70.30 | 33.11 | LN-AuNPs more effective |
| MDA-MB-231 | 1 | t-test | 0.5128 | 100.55 | 102.87 | LN more effective |
|  | 10 | t-test | 0.0004 | 93.71 | 83.86 | LN-AuNPs more effective |
|  | 100 | t-test | 0.0012 | 80.30 | 57.05 | LN-AuNPs more effective |
|  | 1000 | t-test | 0.0149 | 34.95 | 24.83 | LN-AuNPs more effective |

**Supporting Table S3.**

**Pairwise Mann–Whitney U test results comparing cytotoxic responses among cancer cell lines treated with LN-AuNPs.**

At each dose level (1, 10, 100, and 1000 µg/mL), pairwise comparisons of % cell viability were performed among the L929, SH-SY5Y, A549, and MDA-MB-231 cell lines. No statistically significant differences were observed (p > 0.05), likely due to sample size constraints (n = 3 per group).

| Dose (µg/mL) | Comparison | Mann–Whitney  U | p-value | Significant?  (p < 0.05) |
| --- | --- | --- | --- | --- |
| 1 | L929 vs SH-SY5Y | 0 | 0.1000 | No |
|  | L929 vs A549 | 0 | 0.1000 | No |
|  | L929 vs MDA-MB-231 | 0 | 0.1000 | No |
|  | SH-SY5Y vs A549 | 9 | 0.1000 | No |
|  | SH-SY5Y vs MDA-MB-231 | 3 | 0.7000 | No |
|  | A549 vs MDA-MB-231 | 3 | 0.7000 | No |
| 10 | L929 vs SH-SY5Y | 0 | 0.1000 | No |
|  | L929 vs A549 | 0 | 0.1000 | No |
|  | L929 vs MDA-MB-231 | 0 | 0.1000 | No |
|  | SH-SY5Y vs A549 | 9 | 0.1000 | No |
|  | SH-SY5Y vs MDA-MB-231 | 9 | 0.1000 | No |
|  | A549 vs MDA-MB-231 | 0 | 0.1000 | No |
| 100 | L929 vs SH-SY5Y | 0 | 0.1000 | No |
|  | L929 vs A549 | 9 | 0.1000 | No |
|  | L929 vs MDA-MB-231 | 6 | 0.7000 | No |
|  | SH-SY5Y vs A549 | 9 | 0.1000 | No |
|  | SH-SY5Y vs MDA-MB-231 | 9 | 0.1000 | No |
|  | A549 vs MDA-MB-231 | 0 | 0.1000 | No |
| 1000 | L929 vs SH-SY5Y | 9 | 0.1000 | No |
|  | L929 vs A549 | 9 | 0.1000 | No |
|  | L929 vs MDA-MB-231 | 9 | 0.1000 | No |
|  | SH-SY5Y vs A549 | 0 | 0.1000 | No |
|  | SH-SY5Y vs MDA-MB-231 | 3 | 0.7000 | No |
|  | A549 vs MDA-MB-231 | 9 | 0.1000 | No |
